# Competition in Public Procurement in the Czech and Slovak Public Health Care Sectors

**DOI:** 10.3390/healthcare8030201

**Published:** 2020-07-07

**Authors:** Juraj Nemec, Matus Kubak, Milan Krapek, Maria Horehajova

**Affiliations:** 1 Faculty of Economics and Administration, Masaryk University, Brno 602 00, Czech Republic; Juraj.Nemec@econ.muni.cz; 2 Faculty of Economics, Matej Bel University, Banska Bystrica 975 90, Slovakia; 3 Faculty of Economics, Technical University, Kosice 040 01, Slovakia; matus.kubak@tuke.sk; 4 Faculty of Business and Managements, University of Technology, Brno 612 00, Czech Republic; milan.krapek@ambis.cz

**Keywords:** public procurement, health care, efficiency, competitiveness, Czech Republic, Slovakia

## Abstract

Sustainability of health financing is a critical issue for all countries, especially now in the COVID-19 period. The final level of achievements of critical public health goals is connected not only with the efforts of the people involved, but also with the availability of funding to cover the costs of the actions needed. One of the “internal sources” providing more resources to cover public health care costs is effective public procurement in the health care sector. According to existing scientific literature, a low rate of competition represents one important factor that has a direct negative impact on the efficiency of public procurement. The aim of our article is to examine the degree of competitiveness of public procurement in the Czech and Slovak health care systems and its impact on the final price of a contract. The research fully attested the findings of those studies carried out so far – the higher the number of tenderers, the lower the final price, even in the Czech and Slovak health sectors. However, the average number of tenderers is only around two and in the Czech Republic for more than half of the tenders only one bid was submitted.

## 1. Introduction

Health spending in developed countries oscillates from approx. 9 to 18% of the Gross Domestic Product (GDP), however, there is no country where this substantial sum fully covers all perceived health care needs. Health finance on the one hand represents a critical factor “endangering” global fiscal sustainability, on the other hand these resources are expected to finance the growing health care needs of the world’s population, as formulated for example by the UN Sustainable Goal 3 “Ensure healthy lives and promote well-being for all at all ages”. Sustainable Development Goals (SDGs) [1] define 13 critical targets for the health care systems of the world, with focus on reducing the global maternal mortality ratio, reducing neonatal mortality, combating the epidemics of AIDS, tuberculosis, malaria and neglected tropical diseases and to combat hepatitis, water-borne diseases and other communicable diseases, reducing premature mortality from non-communicable diseases and prevention and treatment of substance abuse. SDGs call for achieving universal health coverage, including financial risk protection, access to quality essential health-care services and access to safe, effective, quality and affordable essential medicines and vaccines for all.

Especially after the COVID-19 crisis, it will not be possible to achieve the planned critical goals without a significant increase of efficiency of health expenditures. For most countries it will be very difficult to add extra (public or private) resources to health care, thus the mobilisation of internal reserves by providers is a “must”, to cope with increased health expenditure needs and decreased health care revenues. There exist numerous mechanisms supporting efficiency increases of health care spending—many countries for example use public private partnership (PPP) schemes (especially in the infrastructure area) to address limited health resources and to increase efficiency of health spending. 

Improving public procurement by health establishments, which is the focus of our paper, certainly represents important factors of increased efficiency of health establishments. Worldwide, goods, services and works amounting to approximately 15% of GDP are purchased in developed countries through public procurement—this means that purchasing goods, services and works may represent 20–40% of health care expenditures, depending on the weight of salaries in the health care bill. Simple calculations suggest that improving the efficiency of procurement in a health care sector by 10% (fully possible for most countries) provides (using data for developed countries) up to an extra 0.5% of GDP to cover health needs. Well realised procurement in combination with effective use of Health Technology Assessment [2] in planning procurement needs makes the potential for savings even higher.

Empirical studies (discussed in the literature review part of this paper) fundamentally and unanimously show that higher competitiveness - the number of tenderers—in public procurement leads to lower final prices. However, for all sectors, the Czech Republic and Slovakia belong to those countries with a really small number of bids per tender. According to the European Commission [3], the number of tenders with only one bidder in 2018 was 50% in the Czech Republic and close to 30% in Slovakia and albeit the situation is slowly improving, both countries are still far behind most EU countries.

To reflect the above-mentioned challenges, the goal of our article is to examine the situation in public health care procurement—to measure the rate of competitiveness and its impact on the final price for both selected countries—the Czech Republic and Slovakia. In the analysis, we proceed according to standard approaches published in international articles as well as by domestic authors and respond to the most frequent questions connected with this type of research:

1) What is the level of competitiveness in public health care procurement in the Czech Republic and in Slovakia?
2) What is the impact of the competitiveness on the difference between the forecasted and final price?
3) What is the impact of the method and selection criterion used on the difference between the forecasted and final price?

By responding to our research goal and defined research questions we do not plan to fill any existing theory gap—as indicated above, the fact that increased competitiveness in public procurement saves public money is well documented by many studies (some of them are discussed in the literature review part of this article). However, we want to enrich the existing knowledge by mapping the real situation in the selected and under-researched region of Central and Eastern Europe, where both selected countries are located, and in an under-researched area. The issue of competitiveness/number of tenders in health care purchasing is very rarely investigated by academic literature probably because of the fact that this problem is not an important issue in developed countries, however, as we document, this problem is really urgent for less developed systems and deserves attention. Fifteen years ago, Vlach et al. [4] published a short report benchmarking the economy of public procurement in Slovak hospitals and found excessive inefficiencies. When the findings were presented to the general public, the director of the least performing hospital responded “Our task is to treat patients, not to procure” (Nemec [5]). However, if a hospital is losing millions by ineffective procurement, access for patients to treatment financed from public resources is restricted.

## 2. Public Procurement in Health Care

The number of papers dealing with acquirement of goods, services and works in the health care sector is relatively large. Perhaps the most comprehensive paper mapping health procurement procedures in Europe is that by Decarolis and Giorgiantonio [6]. Their paper analyses the public procurement of orthopedic implants and related medical devices in Europe. Using the data covering the universe of EU public tenders for the period 2009–2014 the authors describe the key empirical features of the market with regard to awarding procedures and contractual forms and discuss the implications for quality, competitiveness, corruption and product innovation.

There are also very many articles dealing with different issues of the public-private interface in the acquirement of goods, services and works for health care needs. Such papers start from a very broad concept of public-private ties and related organisational strategy, managerial, and policy implications [7].

It is necessary to also mention the group of authors who try to introduce and analyse the public-private partnerships as the tool to involve the private sector in developing, financing and providing health infrastructure and services and related critical organisational strategy, management and policy implications [8]. More and more countries in the world are using public-private partnerships to acquire public sector infrastructure and services [9] (however, this is not the case of either of the investigated countries whose PPPs are connected with infrastructure and local communal services only [10]). The concept of PPP is closely related with public procurement, which is the focus of our paper. However, under PPP the complex set of issues needs to be examined and managed—the public-private interface is much more comprehensive compared to procurement, where the goal is just to assure that public bodies purchase in effective and efficient (economical) ways. Torchia et al. [11] delivered in their paper the literature review of 46 articles on this topic. Their findings suggest that although PPPs are used to address internationally emerging public health issues, questions as to their actual effectiveness, efficiency and convenience, still remain problematic. One of the key words for them is “partners”, the other two of efficiency and effectiveness—these factors are also critical for a much more simple public procurement process (which should also not be just about competition—see any of the current articles on sustainable procurement—for example Walker et al. [12]).

Many authors focus on the issue and importance of contracts and contract management. Essig et al. [13] for example try to advance the understanding of performance-based contracting, other authors deal with more concrete aspects of contract and contract management and document that low quality contract management is an important source of inefficiencies (studies focusing directly on the Czech and Slovak environment from this point of view exist—e.g., [14,15,16].

However, the focus of this paper is much narrower. The authors deal exclusively with one small, albeit really important element of the public-private interface in the acquirement of goods, services and works for health care needs as documented by this text, namely competitiveness and its impact on the final price of the realised tender. The authors are aware that this single element does not work independently from other success-failure factors, but they also feel that an in-depth evaluation of the particular elements of a large system is beneficiary and necessary to obtain a full picture.

According to the findings of the scientific literature, a low rate of competitiveness has a direct impact on the efficiency of public procurement (hereinafter referred to as ‘procurement’), which is most often assessed by comparing the estimated value of the contract with the tender price. As one of the most frequently cited authors in this area, Gupta [17] found that six–eight tenderers are needed to achieve the highest competitiveness and that any further increase in the number of tenderers does not affect the final price (the author analysed the area of highway infrastructure construction in Florida from 1981 to 1986, the total sample consisted of 1937 tenders). Gupta [17] calculated that increasing the number of tenderers from two to eight represented achieving an additional saving of 12–14% on average (when analysing data adjusted for extreme values, where the highest competitiveness was achieved with six tenderers, the increase in the number of tenderers from two to six resulted in additional savings of 9 to 10% on average).

Brannman et al. [18] investigated the effect of competitiveness on price, utilising data from three different areas: timber sales (where oral auctions were additionally frequent), oilfield sales (with very inaccurate estimates of oil content) and government bond sales—the findings of this research can also be applied to procurement. The work identified the determinants that affect the final price of the auction (type of goods/services, characteristics of bidders, characteristics of the industry, type of auction used and number of bidders). In four out of six types of auctions, the highest competitiveness was achieved with seven to eight bidders and with five bidders in the other two types of auctions.

Gomez-Lobo and Szymanski [19] investigated the relationship between costs and numbers of bidders for U.K. local authorities’ refuse collection contracts. They found that a higher number of bids is associated with a lower cost of service. Similar results were delivered by Cubbin et al. [20] for the same service.

Atsushi [21] focused on procurement in 26 developing countries, with data from 214 cases from April 1999 to March 2005. The author found that an incrementation in the number of tenderers by an average of 1% brings savings of 0.2% and the state of highest competitiveness was achieved with a participation of eight tenderers. When the number of tenderers is more than eight, the cost-cutting effect is significantly lost.

Gineitienė and Šerpytis [22] analysed procurement of technically identical and standardised goods. The results were similar to those of the previous authors and ergo, with the incrementing number of tenderers, the price is lower than the estimated price. The authors additionally found that with an incrementation from one tenderer to two, significant savings were achieved (for some goods even higher than 10 or 20%).

Ilke et al. [23] analysed competitiveness in procurement in Turkey between 2004 and 2006, utilising data from 90,089 tenders. The results were similar to the abovementioned authors, albeit each supplemental tenderer reduced the resulting price on average by 3.9%. The authors additionally found that tenders with a higher forecasted value would attract more tenderers on average, and hence the size had a direct effect on the number of tenderers.

Kuhlman and Johnson [24] analysed constructions of U.S. highway infrastructure. Their findings, unlike those of e.g., Ilke et al. [23], show that the size of the procurement does not affect the number of tenderers, but substantiated the disproportionate relationship between the number of tenderers and the resulting price. Furthermore, they found that the failure to disclose the forecasted value of the contract (calculated but kept secret) incremented the uncertainty among tenderers and resulted in a lower price compared to those countries that published the forecasted value of contracts.

The research on the level of competitiveness and its impacts on the procurement results is also relatively frequent in the conditions of the Czech and Slovak Republics. Soudek and Skuhrovec [25] analysed procurements of homogeneous products in the Czech Republic, namely electricity and natural gas. The advantage of the analysis of homogeneous goods is the facileness of comparability with the market price, which the authors obtained from the short-term commodity exchange of OTE a.s. electricity and gas in the Czech Republic. The authors pointed out several important findings in their work, according to which, contracting authorities regularly overestimate the forecasted value of the contract and ergo their price forecast does not correspond to the current market price. Of interest is that the greatest impact on the final price is due to the chosen method; the utilisation of open tender decremented the achieved price on average by 7%. On average, each supplemental tenderer decremented the final price by 1%. The impact of the number of tenderers on the resulting price was statistically insignificant in the case of gas supply, which may also be related to an insufficient amount of data. A consequential contribution of Soudek and Skuhrovec [25] is the comparison of the market price, the forecasted value of the contract and the resulting price, and found that despite the overestimation of the forecasted value of the contract, the distinctions between the resulting price and the market price decrease over time thereby bringing savings to the public finances. On average, four tenderers participated in electrical energy procurements and 3.3 tenderers in natural gas procurements.

Pavel [26] analysed the impact of competitiveness on the price of construction of road and railway transport infrastructure in the Czech Republic in the period 2004–2009. In his work, he utilised the relationship of the forecasted value of the contract and the tender price and found that, on average, each additional tenderer reduced the resulting price by 3.27%. Similar to Soudek and Skuhrovec [25], he found that the utilisation of restricted tender increases the price, in this case by an average of 11.56% of the forecasted contract value. An important finding in Pavel’s work [26] is the fact that, as the number of offers increments, the tender price decrements, but the share of the five largest construction companies in the total volume of contracts does not decrement. The author here points out that a vigorous competitive environment forces the top five companies in the market to lower their prices to maintain their market share and win the contract. In his other study Pavel [27] investigated the relationship between the number of bidders and the final price for the road building industry. The results are similar—the calculated extra price decrease for a second tenderer was 7.9%, for a third 7%, for a sixth only 4.5% and the competition effect disappeared for PP with ten bidders. The summary of findings of this author on the efficiency of public procurement is provided by Pavel [28].

Another work that examined in more detail the relationship between competitiveness and the resulting price is the analysis of Šípoš and Klátik [29], where the authors focused on subliminal, below and above threshold procurements in Slovakia in 2012. The analysis focused not only on competitiveness, but also on the impact of utilising e-auctions and of the procedures used. The authors found that as the number of tenderers incremented, the resulting price of tenders declined on average (compared to the forecasted value of the contract), but the relative savings when two tenderers participated were higher than when three and four tenderers participated. The highest savings were achieved with five or more tenderers. According to the authors, using e-auction achieved additional savings of 5%.

Sičáková-Beblavá, Klátik and Beblavý [30] also examined the effect of competitiveness on the final price. The authors examined the impact of e-auction on the efficiency of procurement in a sample of 725 cases between 2008 and 2010 in 32 public sector organisations in Slovakia. The authors’ findings point to the positive impact of e-auction on the efficiency. According to their findings, the first bid reduces the price by an average of 4% and each subsequent bid reduces it by 84% of the precedent reduction. According to the authors, three bidders should be enough to achieve 10% savings and, according to the authors, savings of up to 20% can be achieved.

Grega [31] in his early study presents very similar findings–when five or more tenderers were involved in the PP, the greatest savings were achieved compared to the forecasted contract value. The author returned to the topic also in other publications. The most detailed and comprehensive analysis is delivered by Grega [32]. The author first calculated the level of competitiveness in the Czech (2008–2013) and Slovak (2010–2014) procurement of goods, services and works. He found that in both countries the highest number of bids was received for works and the lowest for goods (somewhat of a surprise, compared to other studies). The calculation of the impact of the number of bidders on the final price was delivered only for Slovakia on the sample of 27,234 procurements between 1 January 2009 and 12 August 2014. His results are similar to others: the calculated price decrease for a second tenderer was 7.7%, for a third 8.9%, for a fourth tenderer 11.75% and the peak 16.9% was achieved in PP with five tenderers. Other papers related to the topic—and with similar findings—by Grega and different co-authors are for example Grega et al. [33], Nemec et al. [34], Nemec et al. [35], Sumpikova et al. [36] 2016 and Grega and Nemec [37,38].

## 3. Methodology

In our research we utilise the most commonly used approach for quantifying the efficiency of PP—comparing the forecasted value of the contract with the final tender price, customarily utilising the lowest price as the criterion. It is a transparent, unpretentious and expeditious way to evaluate, even though its use is associated with certain problems. The main limit of this method is a phenomenon called fictitious savings - setting the higher forecasted value of the contract is propitious for the contracting authority because it can achieve higher (albeit fictitious) savings. According to some authors, the tender price should be compared to the market price (Soudek and Skuhrovec [25], Millet et al. [39] and others) and not the forecasted price. The quandary is that the market price is not always affordable and clearly determinable, especially for services and construction work. For example, sizably voluminous-scale construction work, information systems or legal and advisory services should not be compared with the price on the market and their comparison, if any, should be viewed with reservation. Utilising the market price as a benchmark is felicitous for homogeneous goods and services purchased on a regular basis, with a precise quality specification (in the case of some products of a generally known quality) and with an adequate number of suppliers—and also for a shorter period of time so that prices are impervious to inflation or technical progress. In our case, due to the abovementioned limits and the sizably voluminous sample size, the only solution was to utilise a simple procedure to compare the forecasted and final price, albeit we are cognizant of its limitations.

The main method is our own primary quantitative research, covering a fully comprehensive and representative sample. When calculating the achieved savings, we processed a complete sample of all procurements in the Czech and Slovak health services for 2019 (all registered PPs for the given period—almost 3000 cases—are covered and their information processed by us). Data on individual PPs were obtained from the national registers of the Public Procurement Office and processed by the authors of this article. We use five variables to make statistics analysis (Table 1). The most important variable is savings. We use the following formula to count it:

$$savings=\frac{final\;price-forecasted\;price}{forecasted\;price}$$

We have omitted the extreme values (savings or overspendings over 60%) that could have been caused by transcription errors or major errors in estimating the forecasted PP price and cannot be caused by regular market competitiveness. To check for possible irregularities caused by a one-year picture we also analysed compete samples of the Slovak health care procurements for the period 2014–2018. These data are not directly used in this study, but confirm that the 2019 situation is fully representative.

To process an extensive set of collected data by quantitative methods we use simple descriptive statistics, and, where appropriate, also correlation and regression. We compare savings and other variables by using both methods. We determinate correlation by using the Pearson correlation coefficient:

$$r_{xy}=\frac{\sum_{i=1}^{n}\left(x_{i}-\bar{x}\right)\left(y_{i}-\bar{y}\right)}{\sqrt{\sum_{i=1}^{n}{\left(x_{i}-\bar{x}\right)}^{2}}\sqrt{\sum_{i=1}^{n}{\left(y_{i}-\bar{y}\right)}^{2}}},$$

where $\bar{x}$ and $\bar{y}$ denote arithmetic means. We use a linear regression model with dependent variable savings (*Y*) and k independent variables (*X_i_*):

$$Y=\beta_{0}+\sum_{i=1}^{k}\beta_{i}X_{i}$$

We also apply a qualitative research method to support the discussion part of this paper. To be able to explain the findings better we realised two interviews with intentionally selected experts. In the Czech Republic we interviewed a top academic expert focusing on efficiency of public procurement. In Slovakia we interviewed a top practitioner, the head of the private consultancy firm in procurement. Thanks to this, both academic and practice perspectives are covered (the number of interviews is intentionally limited to two, because the goal of these interviews was to provide the needed specific explanation of purposes for our negative findings).

## 4. Results

We present descriptive statistics of variables in Table 2. Variable savings is limited to interval from −0.6 to 0.6. Variable bids is transformed. We determine the value of eight as the maximum because values higher than eight appear rarely. There are 474 public procurements with missing variable selection criterion because the Slovakia database does not include this information. This is why we analyse selection criterion in the context of the Czech selection procedure only.

**Figure 1:**
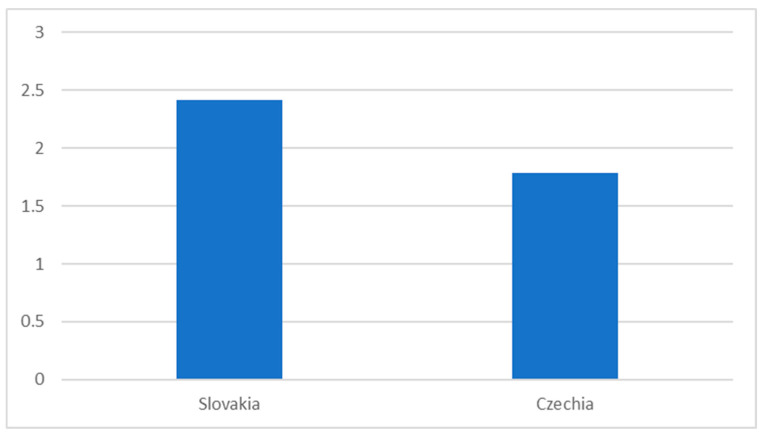
Competitiveness in public procurement in health care in the Czech Republic and Slovakia, 2019.

The data about the average number of offers submitted are very similar for the Czech Republic and Slovakia (and similar to the general pattern of low competitiveness in procurement for both countries). The average number of bids is below two for the Czech Republic and slightly over two in Slovakia (Figure 1).

**Figure 2:**
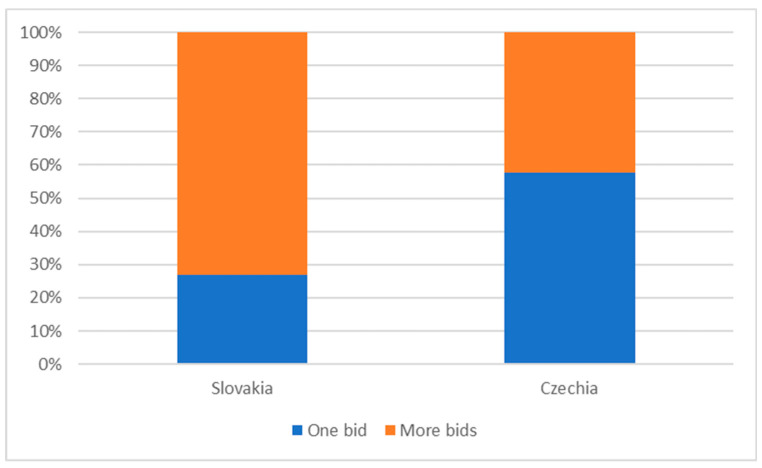
Shares of public procurement with one bidder, 2019.

Given the low competitiveness, procurement in the Czech and Slovak health sectors is characterised by a high proportion of tenders with one tenderer—see Figure 2. The situation in Slovakia is somewhat better, but still approximately one fourth of cases in the monitored period were awarded without any real competition between tenderers (however, in the most expensive field of procurement of medical technology, this figure is almost 60%). The situation in the Czech Republic is critical.

**Figure 3:**
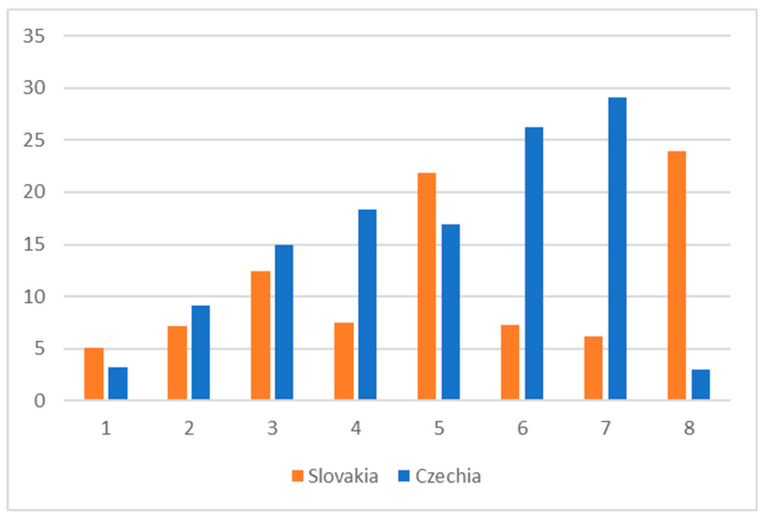
Average difference between forecasted and final price - savings in %, 2019.

When calculating the savings rate (the distinction between the forecasted and final price), as already mentioned, we decided to omit from the analysis all extreme values (over 60% of difference between the forecasted and final price), where the difference could be due to inadequate valuation of the subject of the contract, obvious typing errors, or improper setting of tender conditions. Using simple descriptive statistics, from the perspective of the whole sample, savings clearly depend on the number of bids—see Figure 3. This figure also shows that because of a low level of competitiveness, the results for tenders with more than three bids are dubious. Due to a too low number of such tenders in the sample, one extreme value changes the picture.

To calculate the relationship between the level of competitiveness and savings we also used Pearson correlation index (Table 3) and linear regression analysis. Regarding the weak but statistically significant correlation in all cases, direct linear causality is confirmed—the number of bids decreases the final procurement price.

Regression models confirm statistical dependence between independent and dependent variables, but their prediction value is limited because of the low level of the determinacy coefficient (Table 4 and Table 5).

**Figure 4:**
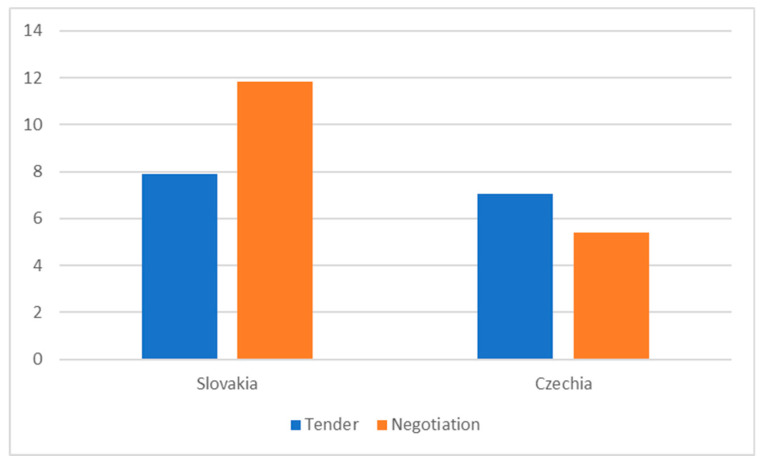
Used method and savings in %, 2019.

We also tried to calculate the relationship between savings and the procurement method used—Figure 4. The results presented by the simple statistics are rather surprising—in the Czech Republic the difference is marginal and in Slovakia non-competitive selection may lead to higher savings. We also tested the sample by correlation and regression analysis; however, for both methods the results are not statistically significant.

**Figure 5:**
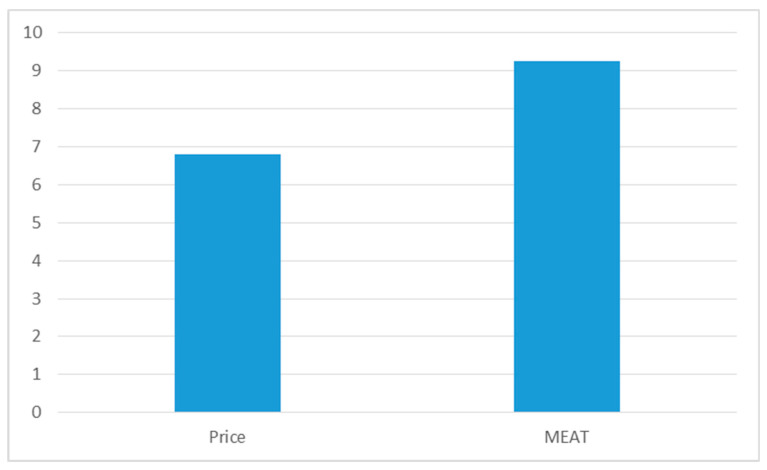
Used selection criterion and savings in %, Czech Republic, 2019.

Finally, Figure 5 shows the level of savings in relation to the selection criterion (lowest price—most economically advantageous bid—MEAT) used—and is calculated only for the Czech Republic, because all procurements in Slovakia in 2019 used the lowest price criterion. We also tested the sample by correlation and regression analysis; however, also in this case the results are not statistically significant for both methods.

## 5. Discussion

Our research used the standard method of comparing the estimated price and the final price of a tender and we tried to relate the calculated savings to the level of competitiveness (number of bids received). As already indicated, this method has important limits. The core problem of this method is a phenomenon called fictitious savings—setting the higher forecasted value of the contract is propitious for the contracting authority because it can achieve higher (albeit fictitious) savings. During our research we confirmed the existence of this phenomenon in our sample. When we analysed the full contract documentation for all Slovak tenders for medical equipment in 2017, the factor of over-estimating the expected contractual value was undoubtedly confirmed (for example, one hospital estimated the price for operating theatre tables at a price of 20,000 EUR, however, the market price for the described quality was approx. 50% less). The existence of fictitious savings moves our results to an even more critical level as it seems that in reality, health care establishments purchase at higher than standard market prices thereby wasting excessive amounts of public resources which would be better used to treat patients.

Another potential problem of our research is the fact that only one year is investigated. However, we are sure that this decision does not limit the validity of our results. The authors have data for all health care procurements in Slovakia for the period 2014–2019 and these data clearly document that using more years for calculations would not help, as the patterns for other years are very similar). Calculation data for the Czech Republic for a longer period would mean wasting time and resources (the need to process more than 10,000 extra data, but with the same final picture).

We also decided to work only with aggregate data for all types of procurement, so goods, services and works are evaluated in one sample. This decision does not impact the results because the level of competitiveness and savings are almost similar for all CPVs (codes of purchased items). It is slightly higher for office purchases, but almost similar for all other procured items (medical technology, support services, medications, etc.).

The fact that two countries are analysed would suggest that a comparative case study method should be more extensively used in this article. However, the results (for non-extreme values) are very similar for both countries and comprehensive comparisons would not help in achieving the main goal of this article. Slovakia achieves a slightly higher level of competitiveness and a slightly higher level of savings, but the differences (especially when taking into account methodological problems – fictitious savings) are just marginal. For both countries, the situation is critical and calls for significant improvements.

The main quandary identified is a very low rate of competitiveness, which limits the efficiency of procurement in the Czech and Slovak healthcare. This fact raises an obvious question—why is the level of competitiveness so limited—are objective (such as a limited size of market) or subjective factors (corruption—active and passive waste) behind it? Two interviews have been organised by the authors to help us to respond to this question, which is not only the specific issue of health care procurement, but the general fact for the whole procurement system in both countries. The response of the Czech academician was very disheartening—according to his opinion the core factor is collusion (corruption) and the size of supplying markets plays only a marginal role. The opinion of the Slovak expert—practitioner—was very similar; however, he mentioned also the factor of excessive bureaucratisation of procurement processes, which limits the will of firms to compete. Also, according to his opinion, the market capacity is not any issue—for well-designed and fair tender the market generates sufficient number of bids.

The opinion of experts that procurement systems in the Czech Republic and Slovakia are the area of collusion agreements and not of fair competition is already confirmed by most recent studies about this topic based on local data and the local situation. Langr [40], in his comprehensive study, directly characterises how the procurement in the Czech Republic functions for most cases. Normally the standard procurement process should include three phases—preparation of tender, tendering and execution. However, in the environment of systemic corruption, two more phases need to be added: phase 0, where collusions are negotiated and the winner and final price are pre-determined and phase 4 where profits are redistributed between the actors involved. The most frequently used tools to limit the competitiveness are a too restrictive description of the object of procurement and too restrictive definition of the qualification criteria.

The opinion of experts and of Langr [40] is supported also by the recent article by Placek et al. [41]. Their paper deals with the issue of overpricing of public procurement in low-performing EU countries and examines a uniquely large sample of public procurement in eleven Central and Eastern European countries. The results indicate that institutional factors have a greater impact on overpricing than individual decisions by the contracting authority. This analysis provides interesting results and also draws attention to behaviour that is not typical of the better established and more advanced EU countries. According to Vesely [42] and some other authors, the source for this situation is, to a large extent, path-dependency—petty corruption was a typical feature of the so-called “socialist” society, and the change of the system opened the space for massive—systemic—large scale corruption, which is tolerated by citizens (Orviska and Hudson [43] or Hunady [44]) and exaggerated by limited accountability and responsibility of politicians and top civil servants (Thijs et al. [45]) and also by a limited managerial culture in the “post-soviet” countries (see for example Spacek and Hornakova [46], Spacek and Pucek [47], Spacek [48] and Nemec [49].

## 6. Conclusions

The goal of this article was rather simple—to analyse and to document the level of competitiveness in Czech and Slovak health care procurement and to assess its impact on the efficiency of health care public procurement results (final price).

From the point of general theory, the processing of data on procurement in the Czech and Slovak public healthcare sectors for the year 2019 fully attested the findings of the studies carried out so far which analysed the relationship between competitiveness in PP and the final price. Even in this sector, a higher number of offers clearly engenders a lower final price. From the data obtained, we are unable to state the optimum rate of competitiveness—most of the abovementioned studies estimated that the optimum number of bids oscillates around 6, whereas in the Czech and Slovak health sectors the average number of bids oscillates around two and any calculations of results for more than three bids are misleading, due to the too low number of cases in the sample.

The core problem found is the fact that the potential of competitiveness is not utilised by health care establishments to obtain better prices (and quality) of purchased goods, services and works. On the contrary, we are able to document large scale waste of resources in the sector, which, according to interviewed experts and existing academic studies is not related to any objective environmental factors, but is the result or consequence of systemic corruption prevalent in the public sectors of both selected countries.

Despite the relatively narrow scope of the investigations, our findings are critical for the future of the health systems in both selected countries (and with high probability in the whole Central and Eastern Europe region and in similar transitional or developing countries). Public health care establishments, which dominate the health sectors in the Czech Republic and Slovakia, realise inefficient procurement processes. The costs are borne by patients, who have to wait for necessary treatment, and by the state, which periodically covers existing hospital debts (for more see for example Bjorkman and Nemec [50].

## Figures and Tables

**Table 1:** Summary of variables.

Variable	Type	Description	
Savings	Real numbers	Percentage of savings (from −0.6 to 0.6): Positive numbers denote savings, minus numbers overresponding	
Bids	Integer	Number of submitted offers (1-8)	
Procurement method	binary	0-Negotiation	1-Tender
State	binary	0-Czechia	1-Slovakia
Selection criterion	binary	0-Price	1-MEAT

Source: authors.

**Table 2:** Descriptive statistics.

Variable	Average	Median	Min	Max	Std. Dev.	Q_0.05_.	Q_0.95_.	Missing Obs.
Savings	7.24	1.30	−59.01	59.75	15.72	−9.16	41.50	0
Bids	1.90	1	1	8	1	1	4	0
Procurement method	0.98	1	0	1	0	1	1	0
State	0.18	0	0	1	0	0	1	0
Selection criterion	0.09	0	0	1	0	0	1	474

Source: authors.

**Table 3:** Correlations between the number of bids and savings.

	Czech Republic	Slovakia	Total
Correlation coefficient	0.269780787	0.116284962	0.243082158
Critical value 5%	0.0418	0.0901	0.0379
Number of observations	2196	474	2670

Source: authors.

**Table 4:** Regression: Czech Republic.

	Coefficient	Standard Error	t-Share	*p*-Value	Significance Level
const	1.04167	0.560161	1.86	0.0631	α = 0.1
bids	3.34971	0.255252	13.12	<0.0001	α = 0.01
Determinacy coefficient: 0.072782					
Adjusted determinacy coefficient: 0.072359					
F(1, 2194): 172.2172					
*p*-value(F): 6.26 × 10^−38^					

Source: authors.

**Table 5:** Regression: Slovakia.

	Coefficient	Standard Error	t-Share	*p*-Value	Significance Level
const	4.96933	1.45567	3.414	0.0007	α = 0.01
bids	1.34075	0.52711	2.544	0.0113	α = 0.05
Determinacy coefficient: 0.01352					
Adjusted determinacy coefficient: 0.011432					
F(1, 2194): 6.469963					
*p*-value(F): 0.011289					

Source: authors.
